# Post-COVID Respiratory Sequelae in COPD: Mucus Plugging, Infectious Complications, and Risk-Stratified Follow-Up

**DOI:** 10.3390/jcm15082890

**Published:** 2026-04-10

**Authors:** Florina Cristiana Lucaciu, Norbert Wellmann, Ana Maria Mihai, Alexandra Sima, Ovidiu Rosca, Madalina-Ianca Suba, Andrada Tarau, Alexandra Bosoanca, Monica Marc

**Affiliations:** 1 Doctoral School, “Victor Babes” University of Medicine and Pharmacy, Eftimie Murgu Square 2, 300041 Timisoara, Romania; florina.lucaciu@umft.ro (F.C.L.); ana-maria.mihai@umft.ro (A.M.M.); andrada.dulf@umft.ro (A.T.); alexandra.bosoanca@umft.ro (A.B.); 2 Clinical Hospital of Infectious Diseases and Pulmonology “Victor Babes”, Gheorghe Adam Street 13, 300310 Timisoara, Romania; ovidiu.rosca@umft.ro (O.R.); madalina.suba@umft.ro (M.-I.S.); marc.monica@umft.ro (M.M.); 3 Department XIII, Discipline of Infectious Diseases, “Victor Babes” University of Medicine and Pharmacy, Eftimie Murgu Square 2, 300041 Timisoara, Romania; 4 Center of Research and Innovation in Personalized Medicine of Respiratory Diseases (CRIPMRD), “Victor Babes” University of Medicine and Pharmacy, Eftimie Murgu Square 2, 300041 Timisoara, Romania; 5 Department of Diabetes, “Pius Brinzeu” Emergency Hospital, 300723 Timisoara, Romania; sima.alexandra@umft.ro; 6 Second Department of Internal Medicine, Faculty of Medicine, “Victor Babes” University of Medicine and Pharmacy, 300041 Timisoara, Romania

**Keywords:** COPD, COVID-19, long COVID, bacterial superinfection, VAP, sepsis, DLCO, pulmonary rehabilitation, follow-up, antibiotic stewardship

## Abstract

**Context/Objectives**: In patients with COPD (chronic obstructive pulmonary disease), SARS-CoV-2 (severe acute respiratory syndrome coronavirus 2) infection represents an overlap of viral injury on a lung already affected by pathological mucus, altered mucociliary clearance, chronic inflammation, and impaired antiviral immunity. **Methods**: A focused narrative review (2020–2025) was conducted using clinical, experimental, and consensus evidence. The evidence was synthesized qualitatively, with priority given to cohort studies, meta-analyses, and mechanism-focused studies with clinical relevance. **Results**: Mucus obstruction (“mucus plugs”) is frequent in COPD (41–67%) and is associated with unfavorable outcomes. COPD also increases the risk of post-COVID respiratory sequelae. Bacterial coinfection at presentation is uncommon (3–5%), whereas secondary bacterial infections are more frequent (14–18%), especially in severe disease requiring intensive care, where VA-LRTI/VAP (ventilator-associated lower respiratory tract infection/ventilator-associated pneumonia) become predominant. Sepsis, whether viral or mixed, reflects disease severity and may contribute to functional decline and susceptibility to reinfections; however, the concept of a post-acute “sepsis legacy” in COPD after COVID-19 should currently be regarded as a clinically plausible but still emerging hypothesis rather than an established COPD-specific outcome. During recovery, acute exacerbation risk rises to 5.6% versus 3.9%, peaking in the first 30 days after severe disease (aHR ≈ 8.14). Persistent dyspnea and reduced DLCO (diffusing capacity for carbon monoxide) suggest ARDS-related injury, tissue remodeling, and microvascular dysfunction. **Conclusions**: In COPD, post-COVID respiratory sequelae result from the interaction of mucus, immunity, and infectious/sepsis-related complications. The first post-discharge month is a critical period requiring careful risk stratification and targeted follow-up.

## 1. Introduction

In the post-pandemic period, the post-acute consequences of COVID-19 remain a major challenge in clinical practice due to the variability of manifestations and their impact on functionality and quality of life. By consensus (Delphi), post-COVID respiratory sequelae have been defined as occurring in individuals with probable or confirmed infection, generally within 3 months of onset, with symptoms persisting for at least 2 months, which cannot be explained by another diagnosis and may vary or recur over time [1].

From a respiratory perspective, follow-up studies have highlighted that impaired DLCO (diffusing capacity for carbon monoxide) and imaging abnormalities can persist for months or years after the acute episode, and a meta-analysis with follow-up of up to 3 years confirms DLCO as one of the most common persistent functional abnormalities [2]. Although longitudinal studies with follow-up of up to 4 years suggest an overall trend toward clinical and functional improvement over time, a subgroup of patients remains with residual changes, including “fibrotic-like” changes, which underscores the importance of structured cardiopulmonary assessment in some hospitalized patients [3,4]. In addition, evidence suggests that pulmonary rehabilitation can favorably influence the course of the disease by improving exercise capacity, lung function, dyspnea, and quality of life in patients with long COVID [5,6].

Patients with COPD (chronic obstructive pulmonary disease) constitute a particular population in the analysis of post-COVID respiratory sequelae, with the underlying disease being characterized by chronic airway inflammation, remodeling, and the presence of excessive mucus. Mucus plugs are increasingly recognized as a relevant clinical element, being associated with a higher risk of exacerbations and a more rapid decline in pulmonary function. At the same time, oxidative stress and mucus hypersecretion can reduce ciliary beating frequency and compromise mucociliary clearance, promoting secretion retention and persistence of inflammation [7,8]. Observational data suggest that, after COVID-19, the rate of exacerbations in patients with COPD may increase [9], supporting the notion that COPD can influence not only the initial severity but also the post-acute evolution and the need for post-COVID follow-up [7,8].

Recovery in patients with COPD can be influenced not only by the initial viral injury but also by bacterial superinfection or coinfection. SARS-CoV-2 can alter the epithelial barrier and immune response, facilitating coinfections and especially superinfections in severe forms, thereby amplifying inflammation and lung injury [9]. Meta-analyses show that bacterial coinfection at presentation is rare, while secondary infections are more frequently encountered during the course of the disease, especially in hospitalized patients, despite the frequent use of empirical antibiotic therapy [10]. When superinfection progresses to systemic dysfunction, respiratory-origin sepsis may occur. Additionally, the conceptual overlap between post-sepsis syndrome and post-COVID respiratory sequelae suggests the possibility of a “sepsis legacy,” with potential implications for medium- and long-term recovery [11]. However, in the specific context of COPD after COVID-19, this should currently be regarded as an emerging conceptual framework rather than an established conclusion, because prospective COPD-specific cohort data remain lacking.

Although the literature on post-COVID respiratory sequelae has expanded substantially, the main unresolved problem in patients with COPD is not merely heterogeneity in definitions, symptom sets, or assessment timing, but the limited ability of existing studies to disentangle overlapping mechanisms and clinical phenotypes [12,13]. In this population, baseline COPD-related abnormalities such as dyspnea, cough, sputum production, frequent exacerbations, chronic airway colonization, and impaired DLCO may already be present before SARS-CoV-2 infection, making post-acute findings difficult to interpret in the absence of baseline phenotyping and objective longitudinal follow-up [12,13,14,15]. As a result, persistent COPD activity, post-viral inflammatory injury, superinfection/sepsis-related consequences, and true post-acute SARS-CoV-2 respiratory sequelae may be grouped under the same “post-COVID” label, thereby limiting causal interpretation and clinical applicability [11,12,13,14,15]. In this context, the key gap is the lack of prospective COPD-specific frameworks integrating a mucus-dominant/exacerbator phenotype, microbiological and colonization status, infectious complications, functional assessment, and longitudinal outcomes [7,8,15,16,17,18,19,20,21,22,23,24].

Accordingly, this review is framed around a central paradox: patients with COPD have a pre-existing airway environment characterized by mucus hypersecretion and impaired mucociliary clearance, yet bacterial coinfection at presentation in COVID-19 is relatively uncommon, while secondary infections emerge later in the disease course [7,8,10,17,18,19,20,21,22,23]. This suggests that the interaction between COPD and SARS-CoV-2 may be temporally dynamic rather than simply additive. We therefore examine whether this interaction remains additive throughout the disease course or evolves into a more synergistic pattern during later stages characterized by mucus retention, bacterial superinfection, respiratory sepsis, and delayed recovery, and we discuss how these mechanisms may inform antibiotic stewardship and risk-stratified follow-up.

## 2. Materials and Methods

### 2.1. Study Design and Objectives

This narrative review is based on the premise that post-COVID respiratory sequelae in patients with COPD should not be understood as a linear sequence of viral injury followed by inflammation and fibrotic-like remodeling, but rather as the result of dynamic interactions among three partially overlapping drivers: mucus/mechanical factors (pathological mucus accumulation and impaired mucociliary clearance), infection/immune factors (altered antiviral responses, bacterial superinfection, and respiratory sepsis), and vascular/remodeling factors (persistent gas-exchange impairment, reduced DLCO, and exercise limitation). The objective of the review was therefore to integrate evidence across these domains and relate them to clinically relevant post-COVID phenotypes and to practical implications for diagnosis, antibiotic stewardship, and risk-stratified follow-up in patients with COPD [2,7,8,10,11,15,16,17,18,19,20,25,26,27].

### 2.2. Search Strategy

A structured search of the literature was performed in PubMed/MEDLINE to identify relevant publications published between January 2020 and March 2025. The electronic search was supplemented by manual screening of the reference lists of relevant systematic reviews, meta-analyses, narrative reviews, and consensus or guidance documents. The search strategy was based on combinations of the following terms: “COPD”, “SARS-CoV-2”, “COVID-19”, “post-acute COVID-19”, “long COVID”, “PASC”, “bacterial coinfection”, “secondary infection”, “superinfection”, “ventilator-associated pneumonia”, “VA-LRTI (ventilator-associated lower respiratory tract infection)”, “sepsis”, “DLCO”, and “pulmonary rehabilitation”. This process identified 100 records. After removal of duplicates and exclusion of non-English or clearly irrelevant publications, titles and abstracts were screened for relevance to the predefined clinical domains of the review, and full-text assessment was performed for publications considered potentially informative for COPD-specific post-COVID respiratory sequelae.

### 2.3. Eligibility and Selection of Evidence

Eligible publications included studies conducted in adult populations, such as prospective and retrospective cohort studies, observational clinical studies, systematic reviews, meta-analyses, and mechanistic studies considered relevant to COPD and post-COVID respiratory sequelae. Additional papers with direct clinical applicability to diagnosis, follow-up, or therapeutic decision-making were also considered. Only publications in English were included. Case reports and studies without direct relevance to COPD, respiratory outcomes, or post-acute sequelae after SARS-CoV-2 infection were excluded. To preserve clinical consistency, the review was limited to evidence specifically related to SARS-CoV-2 infection. Because of the narrative design, not all screened studies were cited individually; when several publications addressed the same question, priority was given to the most clinically relevant, methodologically informative, and up-to-date evidence.

### 2.4. Data Synthesis

The selected literature was synthesized qualitatively, and no de novo meta-analysis was performed. Priority was given to studies with high clinical relevance, including cohort studies, meta-analyses, and mechanism-oriented articles that could help clarify the relationship between COPD-related airway pathology and post-COVID respiratory outcomes. Given the narrative design of this review, the aim was not to provide an exhaustive systematic review of all available studies, but rather a structured, clinically focused synthesis of the most relevant evidence. Heterogeneity in study design, follow-up duration, and outcome definitions was taken into account during interpretation, and these methodological constraints are acknowledged in the main text and summary tables. Regarding the use of generative AI, during manuscript preparation, the authors used ChatGPT v5.4 (OpenAI, San Francisco, CA, USA) exclusively to improve the manuscript’s language and readability. The tool was not used to generate or alter data, perform analyses, interpret results, or produce scientific conclusions. All scientific content, interpretations, and conclusions are the original work of the authors.

## 3. Results

### 3.1. Why COPD Modifies the Severity and Recovery in COVID-19 (Mucus/Barrier, Immunity, Microbiome)

Recent literature describes COPD as a heterogeneous disease in which airway inflammation results from complex interactions between structural and immune cells, with damage predominantly affecting the small airways (peribronchovascular remodeling/fibrosis) and the alveolar walls (emphysema) [28]. This heterogeneity is reflected clinically by different phenotypes (predominant dyspnea vs. persistent productive cough; “frequent exacerbator” vs. “non-exacerbator”) and by distinct exposures or etiologies (smoking, biomass exposure, and air pollution) [28]. In the context of SARS-CoV-2 infection, COPD may influence both the initial severity of disease and post-acute recovery through three clinically relevant mechanistic axes: excessive mucus production and mucus obstruction; epithelial barrier vulnerability with impaired mucociliary clearance; and chronic inflammation associated with a reduced antiviral immune response, occurring in the setting of microbiome alterations and colonization patterns that may complicate the distinction between colonization and active infection [7,8,28].

Mucus obstruction, including visible mucus plugs on CT (computed tomography), is common in patients with COPD and has been associated with an unfavorable prognosis, identifying a subgroup with a mucus-dominant airway phenotype that may be more vulnerable to post-viral injury and delayed recovery [7,16]. From a mechanistic perspective, oxidative stress and persistent inflammation may impair ciliary function and alter mucus rheology through dysfunction of ion channels involved in mucociliary transport, such as the CFTR (cystic fibrosis transmembrane conductance regulator) and the large-conductance calcium-activated potassium channel (BK), thereby promoting secretion retention and persistent local inflammation [8,17]. COPD is also characterized by chronic inflammation accompanied by immune dysregulation, including abnormalities of both innate and adaptive immunity, impaired macrophage phagocytosis, and diminished antiviral interferon responses. These abnormalities appear to be more pronounced in the frequent exacerbator phenotype and may contribute to increased susceptibility to viral infections and delayed resolution of inflammation [18,19,20]. Experimental data obtained from the human bronchial epithelium support this concept, showing that cells derived from patients with COPD may sustain higher SARS-CoV-2 viral loads than cells from healthy individuals and may exhibit a more intense inflammatory response together with a blunted interferon response, thereby amplifying tissue injury [21]. In a complementary lung tissue study, antiviral biomarkers in alveolar macrophages, particularly IRF-3 (interferon regulatory factor 3)-related signaling, were associated with reduced lung function and the exacerbation phenotype in COPD [22]. At the population level, meta-analytic data support an association between pre-existing COPD and a higher risk of persistent post-COVID symptoms [14]. In parallel, the respiratory microbiota in COPD shows compositional alterations, particularly in beta diversity, with potential implications for culture interpretation and for the risk of superinfection in severe clinical settings [29].

Taken together, these findings provide a coherent explanation for the clinical observation that patients with COPD may experience a more difficult post-COVID recovery, including an increased risk of exacerbations after a viral episode. The main COPD-related determinants, their potential clinical implications, and selected methodological strengths/limitations of the key supporting studies are summarized in Table 1.

**Critical Appraisal**. Although mucus plugs on CT are associated with worse outcomes and higher mortality in COPD, current evidence does not establish whether they are causal pathogenic drivers or predominantly markers of more severe underlying disease [7,16]. In the setting of COVID-19, this distinction is particularly important because mucus plugging may contribute to impaired mucociliary clearance, secretion retention, superinfection risk, and delayed recovery [8,10,17,21]. However, available studies do not allow firm conclusions regarding causality, and they do not distinguish the effects of mucus plugs themselves from those of baseline structural lung damage, chronic inflammation, or pre-existing functional impairment. Thus, the current literature supports an association, but not a definitive mechanistic hierarchy. Longitudinal studies combining imaging, baseline phenotyping, microbiological and virological profiling, and mucus-targeted interventions are needed to clarify this issue.

**Table 1:** Key COPD-related determinants in post-COVID respiratory sequelae: clinical relevance and methodological appraisal of supporting studies.

No.	First Author (Year) [Ref.]	Study Design	COPD Determinant	Key Findings	Post-COVID Clinical Relevance	Methodological Strengths/Limitations
1	Johansen et al. (2022) [21]	Experimental preclinical in vitro study using primary human bronchial epithelial cells	SARS-CoV-2–epithelium interaction	Higher viral load, lower IFN, and increased inflammation in COPD cells	Greater initial severity and residual inflammation, with an increased risk of sequelae	Clinically relevant use of primary human bronchial epithelial cells; however, the model does not fully reproduce the chronic inflammatory and smoke-exposed background of COPD airways, which may limit extrapolation to in vivo post-COVID responses
2	Diaz et al. (2023) [16]	Retrospective observational cohort study	Mucus plugs (airway obstruction)	Dose-dependent increase in mortality with greater mucus-plug extent; aHR 1.15 for 1–2 segments and 1.24 for ≥3 segments versus no involvement	High mucus burden identifies a high-risk phenotype; closer follow-up and optimization of secretion and exacerbation management	Large sample size and standardized CT-based image interpretation strengthen the clinical relevance of the findings; however, the study cannot distinguish acute from chronic mucus plug formation and does not include microbiological correlation
3	Baraldo et al. (2024) [22]	Cross-sectional case–control study	Altered antiviral signaling (IRF-3 in alveolar macrophages)	Lower IRF-3 associated with lower FEV1 and more frequent exacerbations	Supports innate immune vulnerability and suggests a potential biomarker of disease severity and slower recovery	Translational relevance through direct lung-tissue assessment and biologically plausible association with lung function and exacerbator phenotype; however, the cross-sectional design and likely limited sample size restrict causal and longitudinal interpretation
4	Kou et al. (2024) [29]	Systematic review and meta-analysis	Microbiome alteration/colonization (dysbiosis)	Changes in composition (beta diversity) predominate; alpha diversity often similar	Supports integrated clinical–microbiological assessment and antibiotic stewardship in COPD patients with post-COVID respiratory symptoms	Higher-level evidence through systematic review and meta-analysis; however, heterogeneity in sampling sites, sequencing methods, study populations, and outcome definitions limits direct clinical comparability
5	Silswal et al. (2025) [17]	Experimental preclinical in vitro study using human bronchial epithelial cell cultures	Mucus hydration (CFTR/BK)	Cigarette smoke increased TGF-β1 and induced CFTR/BK dysfunction, with reduced ASL and CBF; some interventions improved mucociliary parameters	Mechanistic basis for viscous mucus and reduced clearance; requires targeted individualized approach to post-COVID secretion-related vulnerability	Strong mechanistic insight into mucus hydration and mucociliary dysfunction; however, the in vitro design may not fully capture the phenotypic complexity and chronic inflammatory background of COPD in vivo
6	Terry et al. (2025) [14]	Systematic review and meta-analysis	Pre-existing COPD as a long-COVID risk factor	Pre-existing COPD associated with increased risk of long COVID (OR ≈ 1.32)	Requires structured follow-up and risk stratification in post-COVID COPD patients	Meta-analytic design strengthens the overall association between COPD and long-COVID risk; however, differences in long-COVID definitions, follow-up duration, and baseline severity across included studies may affect pooled interpretation
7	Kim et al. (2025) [30]	Retrospective observational cohort study	Post-recovery exacerbation risk (AECOPD)	AECOPD: 5.6% vs. 3.9%; HR 1.45 (95% CI 1.09–1.92); severe AECOPD: aHR 8.14 (95% CI 3.32–19.97) within the first 30 days after severe COVID-19	Supports early post-recovery surveillance, ideally at 4–12 weeks (earlier in high-risk patients)	Clinically relevant cohort data on post-recovery outcomes; however, the retrospective observational design remains vulnerable to residual confounding and variation in baseline COPD severity
8	Li et al. (2025) [15]	Longitudinal observational cohort study	Functional sequelae (diffusion and ventilatory impairment)	Long COVID associated with persistent symptoms and functional decline (incl. DLCO)	Monitor spirometry ± DLCO; refer to rehabilitation if limitation persists	Longitudinal follow-up strengthens relevance for persistent functional impairment; however, interpretation is limited when pre-infection pulmonary function data are unavailable, making attribution to post-COVID injury less certain

### 3.2. Bacterial Infectious Complications in COVID-19 in Patients with COPD: Coinfection, Superinfection, VAP, and Sepsis—Implications for Antibiotic Stewardship

In COVID-19, distinguishing bacterial coinfection at presentation from superinfection acquired during hospitalization is essential because these entities differ in frequency, timing, prognostic significance, and implications for antibiotic therapy [10]. In patients with COPD, chronic airway colonization, excessive mucus production, and impaired mucociliary clearance may further complicate this distinction by (i) masking active infection, (ii) predisposing to superinfection during severe viral pneumonia, and (iii) making microbiological interpretation more difficult because colonization may overlap with true infection.

Available studies consistently show a discrepancy between the relatively low frequency of bacterial coinfection at presentation and the high rate of empirical antibiotic use in patients with COVID-19 [10,23]. Meta-analytic data indicate that bacterial coinfection is uncommon at the time of presentation, whereas secondary bacterial infections occur more often during hospitalization, particularly in severe disease [10,23]. This distinction is clinically important in COPD, where cough, sputum production, and baseline airway colonization may lower the specificity of symptoms suggestive of bacterial infection and may favor unnecessary antibiotic exposure if microbiological and biomarker data are not integrated carefully. Secondary bacterial infections are particularly relevant in hospitalized patients with severe COVID-19 and have been associated with longer hospital stay and increased mortality [31]. In mechanically ventilated patients, VA-LRTI/VAP are frequently reported, often with a predominance of Gram-negative pathogens, especially *Pseudomonas aeruginosa*, *Enterobacter* spp., and *Klebsiella* spp. [32,33]. Beyond the acute pulmonary impact, severe superinfection may progress to systemic dysfunction and sepsis. In cohort studies using Sepsis-3 criteria, sepsis in hospitalized patients with COVID-19 has been associated with severe prognosis, and some cases were classified as viral or mixed rather than purely bacterial [34,35]. This point is important because it further supports the need for microbiological confirmation whenever feasible and cautions against equating all sepsis in COVID-19 with bacterial infection.

An important interpretive limitation is that much of the epidemiologic evidence on bacterial coinfection, secondary infection, and empirical antibiotic use in COVID-19 derives from general COVID-19 cohorts rather than from COPD-enriched populations [10,23,27,36]. Therefore, pooled estimates of bacterial coinfection at presentation and secondary infection during hospitalization should not be assumed to apply directly to patients with COPD, who have higher baseline airway colonization, altered mucociliary clearance, chronic respiratory symptoms, frequent prior antibiotic exposure, and a distinct inflammatory milieu [7,8,17,18,19,20,21,22,23]. This distinction is especially relevant for antibiotic stewardship, because both under-recognition of true bacterial complications and overuse of empirical antibiotics remain plausible risks if general COVID-19 data are extrapolated to COPD without adequate microbiological and clinical contextualization.

**Critical appraisal of antibiotic decision-making.** The low prevalence of bacterial coinfection at presentation in COVID-19 would generally argue against routine empirical antibiotic treatment [10,23]. However, in patients with COPD, this principle is more difficult to apply in practice because the clinical stakes of missing an early bacterial process may be high, while baseline sputum production, chronic colonization, structural lung disease, and impaired mucociliary clearance may reduce the specificity of symptoms and microbiological findings [7,8,17,18,19,20,21,22,23]. Thus, the key clinical question is not simply whether antibiotics should be restricted or broadened, but under which conditions each strategy is justified. The available evidence supports antibiotic restraint at presentation in the absence of convincing indicators of bacterial infection, but it also supports a lower threshold for empirical coverage in selected high-risk scenarios, such as severe pneumonia, ICU admission, mechanical ventilation, sepsis, marked inflammatory response, or strong suspicion of bacterial superinfection [10,23,27,28,29].

Biomarker-guided strategies, particularly those based on procalcitonin, may help reduce unnecessary antibiotic exposure and improve reassessment decisions, as summarized in Table 2 [36,37]. Nevertheless, the evidence remains stronger for reducing antibiotic use at the population level than for guiding decisions specifically in patients with COPD and comorbid structural lung disease. In such patients, chronic airway inflammation, colonization, the frequent exacerbator phenotype, and altered baseline respiratory symptoms may complicate interpretation, so procalcitonin should be used as an adjunct rather than as a stand-alone rule [7,8,14,36,37]. Therefore, the most defensible approach in COPD appears to be an integrated one combining severity assessment, radiologic context, microbiology, biomarkers, and early reassessment/de-escalation, rather than either systematic antibiotic restraint or systematic broad empirical coverage.

The relevance of these complications extends beyond the acute phase. Survivors of sepsis may develop a post-acute syndrome—often referred to as “post-sepsis syndrome” or “sepsis legacy”—characterized by functional decline, vulnerability to reinfections, and increased readmission risk [31,32]. In the context of post-COVID COPD recovery, this framework is clinically plausible and conceptually useful, but the supporting evidence remains indirect and limited, being derived mainly from broader sepsis literature, mechanistic reasoning, and non-COPD-specific COVID-19 observations rather than from prospective COPD-focused cohorts. Therefore, “sepsis legacy” should currently be interpreted as an emerging hypothesis rather than an established COPD-specific consequence of COVID-19. In parallel, prolonged hospitalization, invasive procedures, and cumulative antibiotic exposure increase the likelihood of antimicrobial resistance, reinforcing the importance of antibiotic stewardship in this setting [33,34,35].

Overall, the evidence supports a pragmatic approach in which antibiotics are not prescribed routinely at presentation in the absence of convincing clinical, microbiological, or biomarker-based evidence of bacterial infection, but are reassessed early and adapted according to disease severity, ICU (intensive care unit) course, and the probability of nosocomial superinfection.

The key studies, their population applicability, and their clinically relevant implications for antibiotic stewardship in patients with COPD are summarized in Table 2.

**Table 2:** Key evidence on bacterial coinfection, secondary infection, ventilator-associated infection, and antibiotic stewardship in COVID-19, with clinical relevance and population applicability for patients with COPD.

No.	Study (Author/Year)	Study Design	Population Applicability to COPD (COPD-Specific vs. General COVID-19 Data)	Definition/Context	Key Results (Rate & Outcome)	Clinical Implications(COPD/Antibiotics)
1	Langford et al. (2020) [10]	Systematic review and meta-analysis	General COVID-19 population; not COPD-specific	Co-infections at presentation vs. secondary infection	Bacterial coinfection at presentation: 3.5%; secondary infection: 14.3%; antibiotics prescribed in 71.9% of patients	Supports restraint in routine initial antibiotic use; however, applicability to COPD should be interpreted cautiously because the pooled data are not COPD-specific
2	Ippolito et al. (2021) [38]	Systematic review and meta-analysis	General ICU COVID-19 population; not COPD-specific	VAP in ICU	VAP incidence 45.4%; mortality 42.7%	Supports ICU vigilance and microbiology-guided, rather than routine, antibiotic escalation
3	Carbonell et al. (2022) [39] & Hessels et al. (2023) [25]	ICU/hospital; PCT-guided strategies	General hospitalized/ICU COVID-19 populations; applicability to COPD is indirect	PCT as rule-out tool & antibiotic reduction	PCT < 0.07–0.3 ng/mL NPV > 90%; algorithm reduces antibiotics by 26.8%	Supports antibiotic reassessment and stewardship; potentially useful in COPD exacerbations, but should be interpreted cautiously because the supporting evidence is not COPD-specific and may be less robust in patients with chronic structural lung disease and baseline airway colonization
4	Calderon et al. (2023) [23]	Systematic review and meta-analysis	General COVID-19 population; not COPD-specific, but clinically relevant to stewardship	Reported co-infections vs. microbiologically confirmed	Co-infection 11% (confirmed 4%); antibiotic use > 60%	Supports the need for biomarkers and microbiological confirmation in COPD
5	Langford et al. (2023) [40]	Systematic review and meta-analysis	General COVID-19 population; not COPD-specific	Prevalence + antimicrobial resistance	Co-infection 5.3%; secondary infection 18.4%; resistance > 60%	Strengthens surveillance: limited initial antibiotics + early de-escalation
6	Murray et al. (2024) [41]	Observational retrospective cohort	Hospitalized COVID-19 cohort; not COPD-specific	Secondary infections in hospitalized COVID & post-COVID	Secondary infections 6.9%; longer stay (2.43×); ICU 9.6%; mortality 2.17×	Supports strict infection control + follow-up for COPD
7	Hedberg et al. (2025) [42]	Multinational observational cohort	General multinational COVID-19 cohort; not COPD-specific	Co-infections by waves/variants	Omicron: 7.9% vs. Alpha 2.0% vs. Delta 3.2%; increased mortality	Stratified approach by epidemiology + severity; reconsider empirical therapy
8	Rouzé et al. (2025) [31]	Multicenter retrospective cohort	Critically ill/mechanically ventilated COVID-19 population; not COPD-specific, but relevant to high-risk COPD patients requiring ICU care	Early co-infections vs. VA-LRTI/VAP	Early co-infection 9.7–14.9%; VAP ~36–44.8%; Gram-negative bacilli	Supports awareness of VAP risk in high-risk COPD patients and the need for reassessment after 4–6 days

### 3.3. Pathophysiological Mechanisms Underlying Post-COVID Respiratory Sequelae in COPD: Alveolar Injury, Remodeling, and Endotheliopathy in Relation to DLCO Impairment

Persistent dyspnea and exercise limitation after COVID-19 likely reflect the combined effects of alveolar injury, residual inflammation, post-infectious remodeling, and microvascular/thrombo-inflammatory abnormalities. These mechanisms are particularly relevant in patients with COPD, in whom ventilatory reserve and DLCO may already be impaired before SARS-CoV-2 infection.

After severe COVID-19, some patients show progressive resolution of radiologic abnormalities, whereas others continue to exhibit post-infectious interstitial lung abnormalities, often with an organizing pneumonia pattern, accompanied by significant functional impairment [43]. To reduce inconsistency in interpretation, multisociety international consensus recommendations support the standardized description of post-COVID, CT abnormalities and their integration with clinical findings in order to distinguish residual post-inflammatory lesions from progressive interstitial lung disease [37]. A longitudinal cohort study reported an overall trend toward improvement in CT abnormalities; however, the subgroup with persistent residual changes remained more symptomatic and continued to show impaired gas transfer and reduced performance on exercise testing [44]. Among survivors of COVID-19-related ARDS, fibrotic-like abnormalities have been described at 6 months in a substantial proportion of cases [45]. In addition, patients with COPD are overrepresented in severe forms requiring ICU admission and mechanical ventilation, leading to an increased likelihood of structural and functional sequelae [46].

At the biological level, residual inflammation and profibrotic signaling, including transforming growth factor beta (TGF-β), may promote post-infectious remodeling and symptom persistence [47]. Immunophenotyping and transcriptomic data suggest the persistence of cellular programs involved in repair and remodeling, as well as differences between predominantly inflammatory and predominantly fibrotic post-COVID phenotypes [40]. In COPD, a higher viral load and a diminished interferon response may amplify epithelial injury and contribute to dysfunctional repair [21], while fibroblast and myofibroblast activation remains a central pathway in fibrogenesis [26]. The microvascular component is also clinically relevant. Available studies indicate that perfusion abnormalities and endotheliopathy/thromboinflammation may contribute to reduced DLCO and persistent dyspnea even when parenchymal changes improve [24,27]. In patients with COPD, this mechanism may be particularly important because DLCO may already be reduced due to emphysema and pulmonary vascular abnormalities; additional post-SARS-CoV-2 microvascular injury may therefore aggravate dyspnea and increase exercise limitation.

**Diagnostic dilemma.** In clinical practice, the interpretation of reduced DLCO after COVID-19 is particularly challenging in patients with COPD when no pre-infection functional data are available. In such cases, a low DLCO cannot be attributed automatically to post-COVID injury because it may reflect pre-existing emphysema, pulmonary vascular disease, prior smoking-related alveolar destruction, or the natural progression of COPD itself [24,27,43,44,45,46]. For this reason, interpretation should rely on pattern recognition rather than on a single isolated value. A disproportionately low DLCO relative to spirometric impairment or FVC, especially when accompanied by persistent dyspnea, exertional desaturation, or exercise limitation, may increase suspicion of additional post-COVID alveolar–capillary or microvascular injury [24,27,44]. Correlation with imaging is also essential: residual ground-glass opacities, organizing-pneumonia-like changes, fibrotic-like abnormalities, or persistent perfusion-related changes may support the interpretation of newly relevant post-COVID damage, whereas a predominantly emphysematous pattern without new post-inflammatory abnormalities may favor a larger contribution from baseline COPD [37,43,44,45]. Therefore, in the absence of baseline DLCO, the most defensible strategy is an integrated one combining symptoms, spirometry, the DLCO/FVC relationship, exercise testing, and CT findings, ideally complemented by longitudinal reassessment to determine whether gas-transfer impairment is improving, stable, or progressively disproportionate over time.

**Figure 1:**
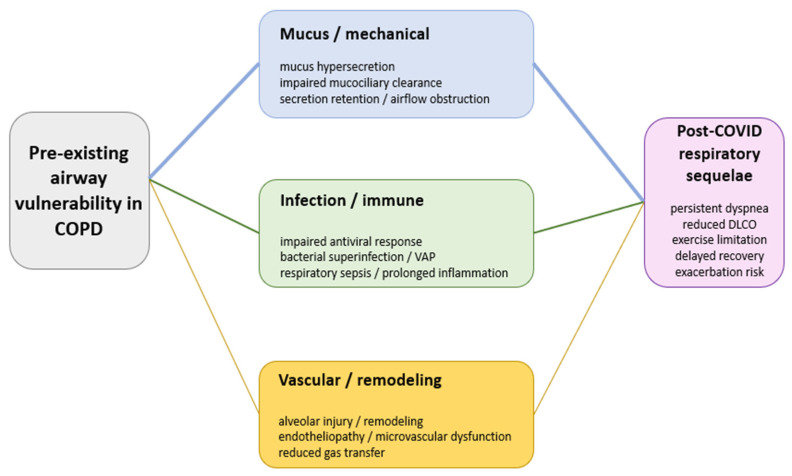
**Simplified conceptual model of post-COVID respiratory sequelae in COPD.** Rather than a single linear cascade, the model presents three parallel pathways linking pre-existing airway vulnerability in COPD to post-COVID respiratory sequelae: mucus/mechanical, infection/immune, and vascular/remodeling. Together, these pathways help explain persistent dyspnea, reduced DLCO, exercise limitation, delayed recovery, and increased exacerbation risk. Variations in line thickness and color intensity were used to indicate the relative importance of the depicted pathways in the COPD population [7,8,10,16,18,19,20,21,22,24,27,29,30,31,32,33,34,35,36,38,41,43,45,46,48].

To improve conceptual clarity, Figure 1 summarizes post-COVID respiratory sequelae in COPD as the result of three parallel and partially overlapping mechanism pathways—mucus/mechanical, infection/immune, and vascular/remodeling—arising from pre-existing airway vulnerability and converging on persistent respiratory sequelae.

**Figure 2:**
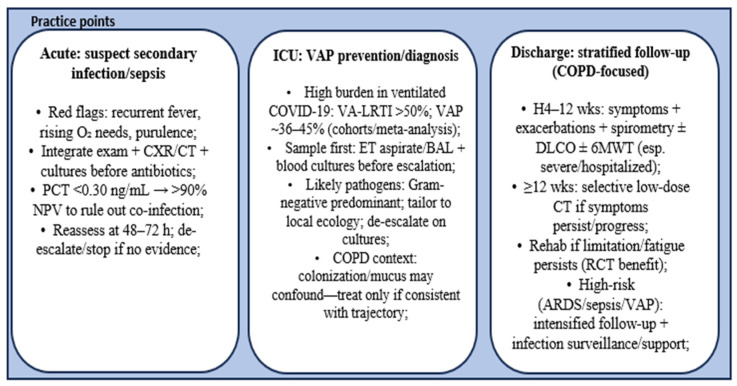
Clinical framework linking infectious complications and post-COVID follow-up in patients with COPD. The figure illustrates how secondary infection, sepsis, critical care complications, antimicrobial stewardship, and respiratory functional assessment may inform post-acute evaluation and follow-up in patients with COPD after COVID-19. Arrows indicate the proposed clinical relationships and workflow direction [7,10,13,25,26,27,29,30,31,32,33,34,35,36,37,38,39,41,45,48,49].

Building on this framework, Figure 2 translates these mechanistic links into pragmatic practice points—supporting early recognition of secondary infection/sepsis, VAP prevention/diagnosis in critical care, and a COPD-focused, risk-stratified follow-up plan (including spirometry, DLCO/6MWT, selective imaging, rehabilitation, and antimicrobial stewardship) to mitigate persistent symptoms and functional impairment.

### 3.4. Diagnosis and Risk Stratification (Acute and Post-Acute)

For patients with COPD and COVID-19, diagnosis must be decision-oriented: in the acute phase, rapid identification of superinfection/sepsis is necessary while avoiding the prescription of unjustified antibiotics; in the post-acute phase, the aim is to recognize sequelae that require targeted monitoring and recovery interventions [50].

**Acute phase (superinfection/sepsis):** In clinical practice, suspicion of superinfection/sepsis is supported by clinical deterioration after a period of stabilization (rapid increase in oxygen requirements, recurrent fever, appearance of purulent secretions, hemodynamic instability, altered mental status), in association with biomarkers and paraclinical investigations. In a cohort study, sepsis in hospitalized COVID-19 patients could be viral, mixed, or, more rarely, strictly bacterial, which underscores the importance of microbiological confirmation whenever possible [34]. According to some studies, procalcitonin can be particularly useful in antibiotic discontinuation/de-escalation strategies for selected patients, as part of a stewardship and clinical reassessment package, with data supporting a reduction in antibiotic exposure without major harm [25,39,51]. In the ICU, the risk of nosocomial pathogens requires appropriate sampling and rapid adjustment of treatment in suspected cases of VAP/VA-LRTI. In patients admitted to the ICU, *Acinetobacter baumannii* was the most frequently isolated pathogen [31,33]. The comparative parameters of coinfections/superinfections and stewardship are summarized in Table 2.

**Post-acute phase (sequelae and triage):** After COVID-19, patients with COPD have shown an increased risk of AECOPD (acute exacerbations of chronic obstructive pulmonary disease), persistent symptoms, and repeated hospitalizations [15,41]. A pragmatic assessment is based on a “minimal package” (symptoms/dyspnea, exacerbations, spirometry, exercise evaluation), supplemented in some cases with DLCO and CT, depending on symptomatology. The multisociety consensus recommends performing a chest CT scan 3 months after infection in patients with persistent/progressive respiratory symptoms, using low-dose protocols (1–3 mSv) for follow-up and standardized reporting [37]. Some studies show that pulmonary rehabilitation can improve exercise capacity and fatigability in selected patients, particularly those with persistent functional limitation after the acute phase; however, the evidence remains heterogeneous across rehabilitation models and patient populations, and access may be limited in routine practice [49,52,53].

### 3.5. Integrated Management: Acute → Discharge → Follow-Up (Emphasis on Continuity and Practice)

To maximize clinical relevance, the management of patients with COPD and COVID-19 must be viewed as a continuum: stabilization in the acute phase (including stabilization of respiratory failure), prevention of complications and sequelae, followed by staged follow-up with clear criteria for investigations and treatment.

**Acute phase (respiratory support and anti-infective management):** In patients with acute respiratory failure, the choice between HFNC (high-flow nasal cannula) and NIV (non-invasive ventilation) depends on the clinical profile of the patient with COPD, including the presence of hypercapnia and/or acidosis, as well as on close monitoring of treatment response, with clear thresholds for escalation. Data from large trials support HFNC as a non-inferior initial option to NIV in selected patients [54]. In addition, the therapeutic approach should avoid prolonged empirical antibiotic therapy in the absence of evidence of bacterial infection. Stewardship strategies such as reassessment at 48–72 h, microbiological confirmation, and the use of biomarkers (procalcitonin) to support antibiotic discontinuation or de-escalation are relevant, given the discrepancy between the frequency of coinfection and the rate of antibiotic use [10,23,25,51,55].

**Prevention of sequelae (in-hospital and post-discharge):** Early mobilization/physiotherapy of hospitalized patients, including those in the ICU, has been associated in some studies with a reduction in long-term physical deficits [56]. Pulmonary rehabilitation programs after discharge (face-to-face or remote) can improve exercise capacity and reduce fatigue, thereby supporting functional recovery in appropriately selected post-COVID patients [49,52,57]. However, access to structured rehabilitation remains uneven across clinical settings, and the available evidence is heterogeneous with respect to program design, intensity, timing, and patient selection [5,6,49,52,57,58]. Therefore, referral to pulmonary rehabilitation should be prioritized particularly for patients with persistent dyspnea, exercise intolerance, fatigue, or measurable functional limitation, rather than assumed to be equally necessary or beneficial for all post-COVID patients with COPD.

**Figure 3:**
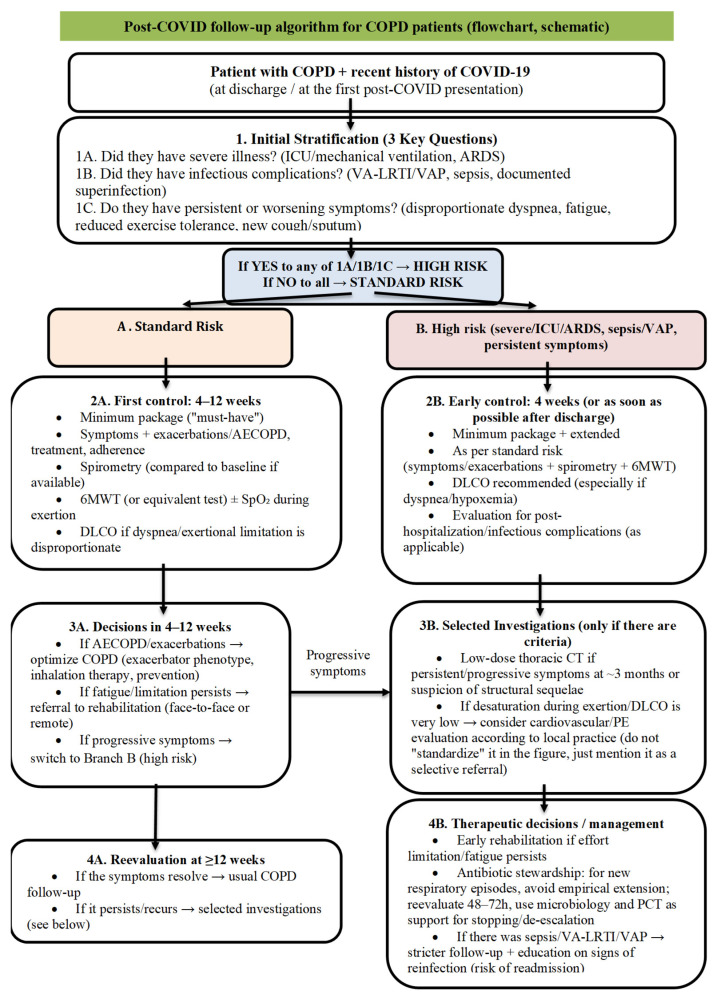
Proposed risk-stratified follow-up framework for patients with COPD after COVID-19. The framework distinguishes standard-risk from high-risk patients and summarizes a pragmatic expert synthesis of the minimum assessment package, indications for selected investigations, and possible follow-up actions. The proposed time windows are based on integration of the available literature, consensus-based follow-up principles, and clinical risk stratification, rather than on COPD-specific prospective trial data directly validating these thresholds. Arrows represent the proposed sequence and direction of the follow-up steps within the risk-stratified framework [25,27,32,34,37,38,41,49,51,52,59].

**Stepwise follow-up for post-COVID patients with COPD:** Follow-up is the central element, as it allows for the early detection of exacerbations, functional deficits, and structural sequelae, and directs patients to pulmonary rehabilitation programs and targeted investigations. As shown in Figure 3, early post-discharge assessment is pragmatically proposed at ~4 weeks for high-risk patients and at 4–12 weeks for standard-risk patients, combining symptom assessment with spirometry and exercise testing (±DLCO). Persistent or progressive symptoms, or high-risk features (severe/ICU course, ARDS, sepsis/VAP), prompt selective imaging and additional evaluation, together with individualized rehabilitation planning and antimicrobial stewardship decisions. These time points should be understood as part of an expert clinical synthesis based on the available literature and consensus-based follow-up principles, rather than as thresholds directly validated by COPD-specific prospective trials [25,27,32,34,37,38,41,49,51,52,59].

## 4. Discussion

This review highlights that recovery in patients with COPD after COVID-19 is influenced by the overlap between pre-existing bronchial vulnerability and complications in the acute phase, especially infectious complications. The COPD background, dominated by pathological mucus, barrier dysfunction, and altered mucociliary clearance, together with impaired antiviral responses, amplifies the initial injury and prolongs inflammation, leading to an increased risk of exacerbations and persistent symptoms. At the same time, the available studies show a gap between the low prevalence of bacterial coinfections at presentation and the frequent use of antibiotics. In addition, superinfections become more relevant during hospitalization, especially in severe cases or in patients admitted to the ICU [10,23,30,31,32,38]. In ventilated patients, VA-LRTI/VAP is frequently reported, with a predominantly Gram-negative etiology (*Pseudomonas aeruginosa*, *Enterobacter* spp., and *Klebsiella* spp.), together with antimicrobial resistance and a direct impact on prognosis and antibiotic stewardship [30,32,38]. Sepsis (viral or mixed, rarely bacterial) represents a major clinical expression of severity and can influence the convalescence period through functional decline and increased susceptibility to reinfections and readmissions (“sepsis legacy”), partially overlapping with the PASC (post-acute sequelae of SARS-CoV-2 infection)/long COVID picture [34,35,36,48]. In the post-acute period, a subgroup of patients remains with dyspnea and low exercise tolerance, frequently associated with low DLCO, reflecting alveolar injury, remodeling, and microvascular involvement. In patients with COPD, these effects may be associated with pre-existing changes [24,27,37,43,44,45,47]. The conceptual integration of these relationships and intervention points is summarized in Figure 1 and Table 2.

### 4.1. From Mechanisms to Post-COVID Clinical Phenotypes in COPD

The background symptomatology of patients with COPD is difficult to distinguish from post-COVID sequelae without objective parameters, which necessitates a phenotype-centered evaluation [12,13,15,60]. The “exacerbator/mucus” phenotype, which is dominated by productive cough, secretion retention, and frequent exacerbations, is supported by data related to mucus plugs and impaired mucociliary clearance. All these characteristics complicate the interpretation of cultures because colonization may overlap with active infection [7,8,16,17,18,29]. The “DLCO↓/exercise intolerance” phenotype highlights the contribution of alveolar injury (especially after severe forms/ARDS (acute respiratory distress syndrome)), post-infectious remodeling, as well as endotheliopathy/thromboinflammation, mechanisms that may contribute to persistent gas-exchange impairment. In patients with COPD, DLCO may already be reduced; thus, an additional decline may have disproportionate functional consequences [24,27,37,43,44,45,47]. Last but not least, the “infectious complications/sepsis legacy” phenotype (superinfections, VAP, sepsis) can hinder recovery by increasing the risk of reinfections and readmissions, supporting closer post-discharge monitoring, especially for subgroups with ARDS/ATI/ventilation/sepsis [32,33,34,35,36,38,48,59].

### 4.2. Clinical Implications (“Practice Points”)

In clinical practice, studies support an algorithm in which suspicion of superinfection is raised at the time of clinical deterioration after a period of stabilization and is confirmed as early as possible through microbiology, when feasible [31,34]. Regarding antibiotic therapy, given the low prevalence of coinfection at presentation, antibiotic treatment requires systematic reassessment at 48–72 h and de-escalation/discontinuation when the probability of bacterial infection is low in order to reduce the emergence of antibiotic resistance [10,23,30,61]. Procalcitonin can be considered a marker for stopping/de-escalating antibiotic therapy in selected contexts, but its interpretation must be integrated clinically and should not be used as a sole criterion. For the prevention of VAP in ventilated patients, rapid adaptation of therapy based on culture results is essential, considering the high incidence and microbiological profile [32,38]. In the post-acute period, the minimum package, which includes symptoms/exacerbations, SpO_2_ (peripheral oxygen saturation) at rest and on exertion, and spirometry, is supplemented in select cases by DLCO and exercise tests when dyspnea is disproportionate or desaturation occurs, while chest CT at 3 months, using low-dose protocols, is reserved for patients with persistent/progressive symptoms [24,27,37]. Pulmonary rehabilitation remains an important option for patients with persistent fatigue, dyspnea, exercise intolerance, or other functional limitations after COVID-19, although referral should be individualized according to clinical need and local resource availability [5,49,52,58].

### 4.3. Screening and Follow-Up Algorithm

The framework proposed in Figure 3 applies a stratified follow-up approach based on the severity of the acute phase and the presence of infectious complications. For patients in the high-risk category, including those with ARDS, ICU admission, invasive mechanical ventilation, superinfection/VAP, and/or sepsis, earlier follow-up and targeted investigations are recommended, together with early referral to pulmonary rehabilitation when functional limitation predominates [31,32,33,34,35,36,37,38,48,49,52,59]. In lower-risk patients, evaluation is performed at 8–12 weeks, with additional testing selected according to persistent symptoms and functional evolution, in order to avoid unnecessary investigations while focusing attention on patients who do not follow the expected recovery trajectory [37].

### 4.4. Knowledge Gaps

The central knowledge gap is not only the lack of standardized definitions for superinfection, VAP, and PASC, but also the absence of prospective COPD-specific studies capable of disentangling overlapping post-COVID mechanisms and clinical phenotypes [12,13]. Current evidence remains fragmented across separate domains—mucus plugging, impaired mucociliary clearance, infectious complications, DLCO impairment, exercise limitation, and rehabilitation outcomes—without an integrated framework linking these features to baseline COPD characteristics and longitudinal recovery [6,7,8,15,16,17,18,19,20,21,22,23,25,39,49,51]. This limitation is particularly important because, in patients with COPD, post-COVID dyspnea, cough, exacerbations, microbiological findings, and gas-exchange abnormalities may reflect pre-existing disease burden, severe acute COVID-related injury, superinfection/sepsis-related consequences, or a true post-acute SARS-CoV-2 phenotype [11,12,13,22,28,30,32,33,34,50]. Therefore, the priority is not only to standardize terminology, but also to develop prospective cohorts with integrated phenotyping that combines baseline COPD profile, mucus/exacerbator status, microbiome or colonization data, infectious events, antiviral response biomarkers, spirometry, DLCO, exercise capacity, and readmission outcomes [6,7,8,15,16,17,18,19,20,21,22,23,25,39,49,50,51].

### 4.5. Limitations

In patients with COPD, the overlap between pre-existing symptoms, baseline functional impairment, and post-COVID manifestations may lead not only to misclassification, but also to incorrect mechanistic attribution when baseline clinical and functional data are unavailable [10,12,13,23,31,32,42]. In addition, some lines of evidence—particularly those related to the microbiome, colonization patterns, and antiviral biomarkers—remain largely associative and still require validation in prospective COPD-specific cohorts with integrated longitudinal assessment [7,8,15,16,17,18,20,21,22,23]. In addition, some conceptual frameworks discussed in this review—particularly the possible role of a post-acute “sepsis legacy” in COPD after COVID-19—remain hypothesis-generating and are not yet supported by prospective COPD-specific cohort data.

Another important limitation is the temporal context of the available evidence. Much of the literature informing this review—particularly data on mucus plugging, superinfection rates, DLCO impairment, and post-acute functional burden—was generated during earlier pandemic waves, when severe disease was more frequent and vaccination coverage was lower. As a result, the applicability of some of these findings to more recent populations exposed predominantly to Omicron-lineage variants, often with prior vaccination or hybrid immunity, remains uncertain. Accordingly, the mechanistic framework and follow-up recommendations proposed here should be interpreted as most applicable to higher-risk or more severe post-COVID COPD trajectories while recognizing that the absolute frequency and intensity of these sequelae may differ in contemporary clinical settings [16,25,30,39,40,41,44,46,47,48,49,50,51,52].

## 5. Conclusions

Patients with COPD represent a vulnerable subgroup in the context of post-COVID respiratory sequelae, as bronchial characteristics such as pathological mucus, a fragile epithelial barrier, and altered mucociliary clearance, together with compromised antiviral immune responses, can exacerbate the initial injury and prolong inflammation. Evidence supports that, although bacterial coinfection at presentation is rare, superinfections acquired during hospitalization, especially in patients with severe forms, those admitted to the ICU, and those requiring mechanical ventilation, are more frequent, are associated with longer hospital stays and an unfavorable prognosis, and require a rigorous approach to antibiotic stewardship.

During the convalescence period, phenotypes characterized by frequent exacerbations, dyspnea or exercise intolerance, and reduced DLCO may reflect a combination of ARDS, pulmonary remodeling, and microvascular impairment, superimposed on pre-existing COPD-related deficits. From a clinical perspective, a staged follow-up, guided by the severity of the acute episode and the presence of superinfection or sepsis, with mandatory investigations and selected tests (DLCO, exercise tests, targeted CT), alongside pulmonary rehabilitation for patients with functional limitations, can optimize recovery and reduce the risk of complications.

As a future priority, the need for specific prospective COPD cohorts with integrated phenotyping (mucus, microbiome/colonization, antiviral response biomarkers) should be emphasized, together with formal evaluation of the role of sepsis and of the proposed “sepsis legacy” framework in the post-acute evolution of the disease.

## Data Availability

The data are encapsulated within this article. Further details can be obtained upon request from either the primary author or the corresponding author.

## Abbreviations

The following abbreviations are used in this manuscript:

AECOPD	Acute Exacerbation of Chronic Obstructive Pulmonary Disease
aHR	Adjusted Hazard Ratio
ARDS	Acute Respiratory Distress Syndrome
ASL	Airway Surface Liquid
BK	Large-Conductance Calcium-Activated Potassium Channel
CBF	Ciliary Beat Frequency
CFTR	Cystic Fibrosis Transmembrane Conductance Regulator
CI	Confidence Interval
COPD	Chronic Obstructive Pulmonary Disease
CT	Computed Tomography
DLCO	Diffusing Capacity for Carbon Monoxide
FEV1	Forced Expiratory Volume in 1 Second
HFNC	High-Flow Nasal Cannula
HR	Hazard Ratio
ICU	Intensive Care Unit
IFN	Interferon
IRF-3	Interferon Regulatory Factor 3
NIV	Non-Invasive Ventilation
NPV	Negative Predictive Value
OR	Odds Ratio
PASC	Post-Acute Sequelae of SARS-CoV-2 Infection
PCT	Procalcitonin
SARS-CoV-2	Severe Acute Respiratory Syndrome Coronavirus 2
SpO_2_	Peripheral Oxygen Saturation
TGF-β	Transforming Growth Factor Beta
VA-LRTI	Ventilator-Associated Lower Respiratory Tract Infection
VAP	Ventilator-Associated Pneumonia
WHO	World Health Organization
6MWT	6-Minute Walk Test
